# Effect of Isoflurane on Neutrophil Phagocytic Function During Pregnancy

**DOI:** 10.1155/S1064744993000237

**Published:** 1993

**Authors:** Penny Clark, A. Joseph Layon, Patrick Duff

**Affiliations:** 1 Division of Maternal-Fetal Medicine University of Florida College of Medicine Gainesville FL USA; 2 Department of Anesthesiology and Medicine University of Florida College of Medicine Gainesville FL USA; 3 Box 100294 University of Florida College of Medicine Gainesville FL 32610-0294 USA

## Abstract

*Objective: * General anesthesia has been considered an independent risk factor for postcesarean infection, but the mechanism for this association has not been delineated. The purpose of this prospective investigation was to determine if phagocytic response of neutrophils was impaired by in vitro exposure to isoflurane, a commonly used anesthetic.

*Methods:* Twelve milliliter venous blood samples were withdrawn from 18 term patients during labor. Neutrophils were separated by Ficoll gradient centrifugation. Aliquots of 2 × 10^6^ neutrophils/ml were exposed to anesthesia using an airtight modular incubator chamber through which a 0.5% isoflurane:50% N_2_O + 50% O_2_ mixture flowed at a rate of 4 l/min for 90 min at 37℃. Neutrophils were assayed for phagocytosis by incubation with *Escherichia coli* conjugated with fluorescein isothiocyanate for 30 min at 37℃. Phagocytosis was assessed by flow cytometry. Neutrophils from the same patient that were not exposed to anesthesia served as controls.

*Results:* The mean percentage of phagocytizing neutrophils in the isoflurane-treated group was 82.8 ± 24 compared to 83.5 ± 22 in the control group. The difference between the two groups was not significant.

*Conclusions:* In vitro exposure to the general anesthetic isoflurane for 90 min does not significantly alter the phagocytic capacity of neutrophils.


Infectious Diseases in Obstetrics and Gynecology 1:98–103 (1993)
© 1993 Wiley-Liss, Inc.

### Effect of Isoflurane on Neutrophil Phagocytic Function During Pregnancy

**Penny Clark, A. Joseph Layon, and Patrick Duff**

*Division of Maternal-Fetal Medicine (P.C., P.D.) and Departments of Anesthesiology and Medicine (A.J.L.), University of Florida College of Medicine, Gainesville, FL*

ABSTRACT

***Objective:*** **General anesthesia has been considered an independent risk factor for postcesarean infection, but the mechanism for this association has not been delineated. The purpose of this prospective investigation was to determine if phagocytic response of neutrophils was impaired by in vitro exposure to isoflurane, a commonly used anesthetic.**

***Methods:*** **Twelve milliliter venous blood samples were withdrawn from 18 term patients during labor. Neutrophils were separated by Ficoll gradient centrifugation. Aliquots of 2 × $10^6$ neutrophils/ml were exposed to anesthesia using an airtight modular incubator chamber through which a 0.5% isoflurane:50% $N_2O$ + 50% $O_2$ mixture flowed at a rate of 4 l/min for 90 min at 37°C. Neutrophils were assayed for phagocytosis by incubation with *Escherichia coli* conjugated with fluorescein isothiocyanate for 30 min at 37°C. Phagocytosis was assessed by flow cytometry. Neutrophils from the same patient that were not exposed to anesthesia served as controls.**

***Results:*** **The mean percentage of phagocytizing neutrophils in the isoflurane-treated group was 82.8 ± 24 compared to 83.5 ± 22 in the control group. The difference between the two groups was not significant.**

***Conclusions:*** **In vitro exposure to the general anesthetic isoflurane for 90 min does not significantly alter the phagocytic capacity of neutrophils.** © 1993 Wiley-Liss, Inc.

KEY WORDS

Host defenses, phagocytosis, leukocyte function, anesthesia

There is a high frequency of postoperative infection following cesarean delivery.[1,2] Of the clinical risk factors for infection, general anesthesia was identified by Green and Sarubbi[3] to be the most statistically significant variable. However, according to Gibbs,[2] general anesthesia was not a consistent determinant for increased rate of postoperative infection. Clearly, the role of anesthesia as a predisposing risk factor for infection following obstetric surgery is not yet well established.

Inhalational anesthetic agents have been shown to suppress the immune system.[4,5] Some agents cause impairment of phagocytic function, an important aspect of nonspecific host resistance.[6–9] Increased susceptibility to bacterial infections has been closely associated with defective neutrophil function. The host depends initially on neutrophils to eliminate invading bacterial pathogens. In obstetric patients, the stresses of surgery and anesthesia during cesarean delivery may diminish the activity of phagocytes, thus predisposing the patient to postpartum infection.

The purpose of the present prospective study was to determine the effect of isoflurane on the phagocytic function of neutrophils collected from laboring patients. Isoflurane is an inhalation anesthetic commonly used in clinical obstetrics in the United States. The technique of flow cytometry

**Address correspondence/reprint requests to Dr. Patrick Duff, P.O. Box 100294, University of Florida College of Medicine, Gainesville, FL 32610-0294.**

Received April 29, 1993
Accepted June 7, 1993

Clinical Study was used to evaluate phagocytosis of fluorescein-conjugated *Escherichia coli* by purified neutrophils after exposure in vitro to isoflurane. Flow cytometry allowed single cell analysis of phagocytic activity.[10–12]

### MATERIALS AND METHODS

The study population consisted of 18 normal term patients between the ages of 18 and 40 years who were in labor when admitted to Shands Hospital, University of Florida, from September to December, 1991. Written informed consent was obtained from each patient in accordance with guidelines established by the Institutional Review Board.

Twelve-milliliter samples of peripheral venous blood were withdrawn into heparinized vacutainer tubes from each of the 18 patients. Granulocytes from whole blood were isolated using a modification of Boyum's Ficoll-Hypaque density gradient centrifugation.[13] Briefly, 3 ml of Histopaque-1119 (Sigma Chemical Co., St. Louis, MO) was transferred into a 15 ml conical centrifuge tube, then layered with 3 ml of Histopaque-1077 (Sigma Chemical Co.). Six milliliters of whole blood was carefully overlayed onto the upper gradient and centrifuged in a swinging bucket rotor at room temperature for 30 min at 700*g*. At the end of centrifugation, a distinct layer of lymphocytes, platelets, and other mononuclear cells settled at the plasma/1077 interphase while a layer of predominantly granulocytes settled at the 1077/1119 interphase. Erythrocytes gravitated to the bottom. Each layer was aspirated; the granulocyte layer was saved and washed 3 times with Hank's balanced salt solution (HBSS; Sigma Chemical Co.) by centrifugation at 200*g* for 10 min. The washed cells were resuspended in RPMI 1640 with 25 mM HEPES and sodium bicarbonate (Sigma Chemical Co.) and counted in a Coulter Counter (Coulter Electronics, Inc., Hialeah, FL). The final suspension contained approximately $2 \times 10^6$ cells/ml. Viability of the granulocytes was determined to be over 95% by trypan blue exclusion.

*E. coli* was labeled with fluorescein 5-isothiocyanate (Sigma Chemical Co.) by the method described by Gelfand.[14] An overnight culture of *E. coli* on trypticase soy broth was heat-killed at 60°C for 30 min, washed 3 times with normal saline, then resuspended in 0.5 M carbonate/bicarbonate buffer, pH 9.5. Fluorescein 5-isothiocyanate dissolved in the same buffer was added to the bacterial suspension at a final concentration of 1 mg/ml and incubated in the dark for 2 h at room temperature. The fluorescein-conjugated bacteria were washed several times and resuspended in HBSS to a final concentration of $1 \times 10^9$ cells/ml. One milliliter aliquots were stored at −80°C and thawed to room temperature immediately before use.

One milliliter suspensions of purified neutrophils were distributed into 35 × 10 mm sterile polystyrene disposable suspension culture dishes (Corning Glass Works, Corning, NY). Neutrophils that were exposed to anesthesia were placed inside an airtight Modular Incubator Chamber (Billups-Rothenberg, Inc., Del Mar, CA) through which a 0.5% isoflurane (Forane USP, Anaquest, Madison, WI):50% $N_2O$ + 50% $O_2$ mixture flowed at a rate of 4 l/min for 90 min at 37°C. The isoflurane vaporizer was calibrated using a Perkin-Elmer mass spectrometer (Perkin-Elmer, Norwalk, CT). Gas samples were taken from the circuit limb containing exhaust gas after chamber equilibration was achieved. For controls, cell suspensions from each patient were incubated at 37°C but not exposed to the anesthetic. The 90 min exposure period was selected because it should be near the maximum duration of exposure to general anesthesia that a patient would encounter during cesarean delivery.

Phagocytosis of fluorescein-conjugated *E. coli* was measured in a reaction mixture containing 0.5 ml cell suspension, 0.1 ml of fluorescent *E. coli*, and 0.4 ml diluted pooled sera. The ratio of neutrophils to bacteria was routinely 1:25 to 1:60. The mixture was allowed to incubate with an end-over-end rotation for 30 min at 37°C. The reaction was terminated by the addition of 3 ml cold 3 mM EDTA in phosphate-buffered saline, then analyzed by flow cytometry.

Flow cytometric analyses were carried out in a FACSCAN (Becton-Dickinson Immunocytometry Systems, Mountain View, CA) equipped with a 15 mW argon laser. The excitation wavelength was set at 488 nm, and green fluorescence was selected by a 530 ± 30 nm band pass filter. Various cell types were discriminated by means of their combined forward and 90° light scatter. The mean channel number of green fluorescence intensity was simultaneously measured. Ten thousand blood cells from each reaction tube were routinely analyzed. Free extracellular bacteria were excluded by their small size. The neutrophils were identified and this population was gated. Consort 30 software (Becton-Dickinson Immunocytometry Systems) was used for data collection and analyses. The number of neutrophils, the percent fluorescent neutrophils, as well as the mean fluorescence per neutrophil were calculated within the gated region. The percent fluorescent neutrophils represented those cells that phagocytized fluorescent *E. coli*. For each patient, the percent phagocytizing neutrophils after in vitro exposure to isoflurane was compared to control cells that were not exposed to isoflurane. Flow cytometric observations were confirmed by fluorescent microscopy (Nikon Optiphot, Nikon, Inc., Garden City, NY).

To ensure that the measured fluorescence was due to actual phagocytosis, we determined the effect of sodium azide, a known inhibitor of phagocytosis. Sodium azide in 100 mM concentration was incorporated into the phagocytosis assay mixture.[15] To further distinguish fluorescence due to internalization of bacteria vs. fluorescence resulting from simple adherence of bacteria to the cell surface of the phagocytes, fluorescence-quenching experiments were performed with trypan blue at low pH.[16] After termination of the phagocytosis reaction, the cells were suspended in cold phosphate-buffered saline with 0.25 mg/ml trypan blue at pH 4.5, then analyzed by flow cytometry.

Diluted pooled sera in the phagocytosis reaction mixture were used for opsonization to improve the efficiency of phagocytosis. Serum was collected from normal pregnant women and frozen in aliquots. Immediately prior to use, an aliquot was thawed and diluted 1:4 with RPMI 1640.

Statistical analysis was performed using the two-tailed paired t-test. $P < 0.05$ was considered significant.

### RESULTS

Different cell types were discriminated by their combined forward light scatter which measured cell size and side scatter which measured granularity. Figure 1 is a representative computer-generated dot plot of the distribution of 10,000 blood cells after neutrophil purification, showing the gated neutrophil population which comprised approximately 71 ± 21% of the total cells enumerated. The percentage of fluorescent neutrophils de-

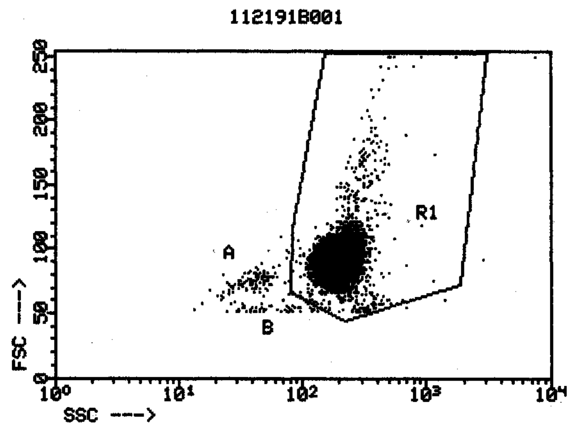

Fig. 1. Representative distribution of 10,000 blood cells in a sample of purified neutrophils from a pregnant patient. Measurement of forward (FSC) and 90° (SSC) angle light scatter discriminated (A) lymphocytes, (B) platelets and erythrocytes, and (R1) neutrophils. The percent fluorescent neutrophils were calculated within the gated region.

termined within the gated region was considered the percent phagocytizing neutrophils.

The optimal ratio of phagocytes to bacteria and length of incubation for routine phagocytosis assay were first determined. Figure 2 demonstrates the rate of increase in percentage of phagocytizing neutrophils with length of incubation, as well as with increasing neutrophil to *E. coli* ratios. Maximal phagocytosis was achieved after 30 min incubation, where approximately 90% of neutrophils were fluorescent. Nonsignificant increases in phagocytosis occurred with longer incubation periods of up to 60 min. The rate of ingestion increased with bacterial concentration and reached saturation at a neutrophil to *E. coli* ratio of 1:25. There was no further increase in the rate of uptake when the number of bacteria was 50, 100, and 150 times the number of neutrophils. Based on these findings, a neutrophil to *E. coli* ratio of 1:25 to 1:60 with 30 min incubation time was routinely used for the phagocytosis assay.

Quenching with trypan blue at pH 4.5 did not alter the percent fluorescent neutrophils after 30 min of phagocytosis, indicating that at the end of this period fluorescent bacteria had been internalized. Sodium azide at 100 mM concentration decreased phagocytosis to 5–7% in both the control and isoflurane-exposed groups.

Panels A in Figure 3 represent two-parameter dot displays of the gated neutrophil population of control cells and isoflurane-treated cells at times 0

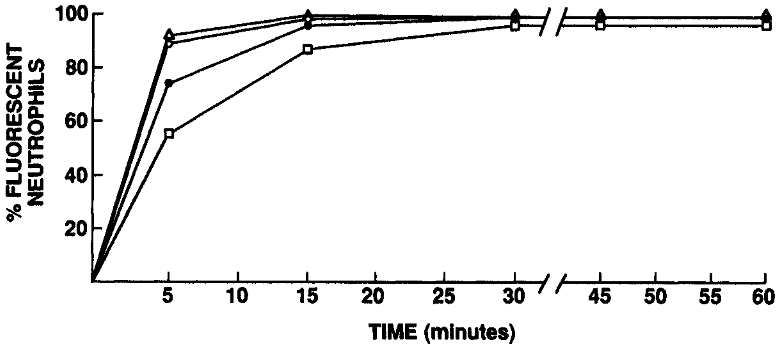

Fig. 2. Rate of phagocytosis and neutrophil to *E. coli* ratio. Neutrophil to *E. coli* (□, 1:4; ●, 1:10; ○, 1:25; △, 1:50, 1:100, 1:150).

and 30 min incubation with fluorescent *E. coli*. The units represent channel numbers or arbitrary values proportional to the intensity of forward and side light scatter. Panels B in Figure 3 represent the corresponding histograms of green fluorescence intensity of neutrophils within the gated region. The mean percent fluorescent neutrophils in this region after in vitro exposure to isoflurane was 82.8 ± 24% compared to 83.5 ± 22% in the unexposed control (NS).

### DISCUSSION

The response of neutrophils to bacterial invasion includes chemotaxis, phagocytosis, oxidative and hydrolytic intracellular killing, and release of lysosomal components. Several studies have demonstrated that inhalation anesthetics depress certain phases of the phagocytic response. Significant inhibition of microbicidal capacity of human neutrophils has been observed with the volatile anesthetic halothane.[6,7] Nakagawara and colleagues[8] studied the effects of halothane, isoflurane, and enflurane on phagocytosis, superoxide production, and intracellular calcium mobilization on neutrophils obtained from healthy adult volunteers. Their results showed that these volatile agents caused a decrease in superoxide production that appeared to be due, at least in part, to inhibition of intracellular calcium mobilization.

Welch[9] reported that enflurane depressed bacterial killing and chemiluminescence only in neutrophils that were stressed by high bacterial challenge, but this inhibition was reversed by exposure to air for 30 min. Earlier investigations demonstrated that halothane, trichloroethylene, diethyl ether, and methoxyflurane inhibited the migration of human neutrophils toward a chemoattractant, casein.[17] Neutrophil chemotaxis was similarly depressed by nitrous oxide and enflurane, but not by enflurane's chemical isomer, isoflurane.[18] In fact, isoflurane stimulated chemotaxis of elicited rabbit neutrophils in vitro.[19] Therefore, different anesthetic agents appear to have different mechanisms by which they affect neutrophil function.

In our experiment, the profound decrease in percentage of fluorescent neutrophils in the presence of an inhibitor of phagocytosis such as sodium azide confirms that the amount of fluorescence in both the control cells and those exposed to isoflurane was due to actual engulfment of the bacteria by phagocytosis. Additional proof of actual internalization of fluorescent *E. coli* was the fact that no further fluorescence quenching was seen with addition of trypan blue in acid pH after 30 min of phagocytosis. The dye would quench fluorescence from bacteria that were attached externally to the cell surface but not those inside the cell.[16]

Our study demonstrates that the ability to phagocytize bacteria remains active in neutrophils of normal pregnant women after exposure to isoflurane. Although this investigation did not examine microbial killing by phagocytes, Welch[9] previously reported that the ability of neutrophils of normal healthy adults to kill *E. coli*, *Klebsiella pneumoniae*, or *Staphylococcus aureus* was not significantly altered when exposed to 1–3% isoflurane for 1 h. Furthermore, no inhibition of microbicidal activity of human neutrophils was observed with 70% nitrous oxide and 30% oxygen, alone, or in combination with isoflurane.

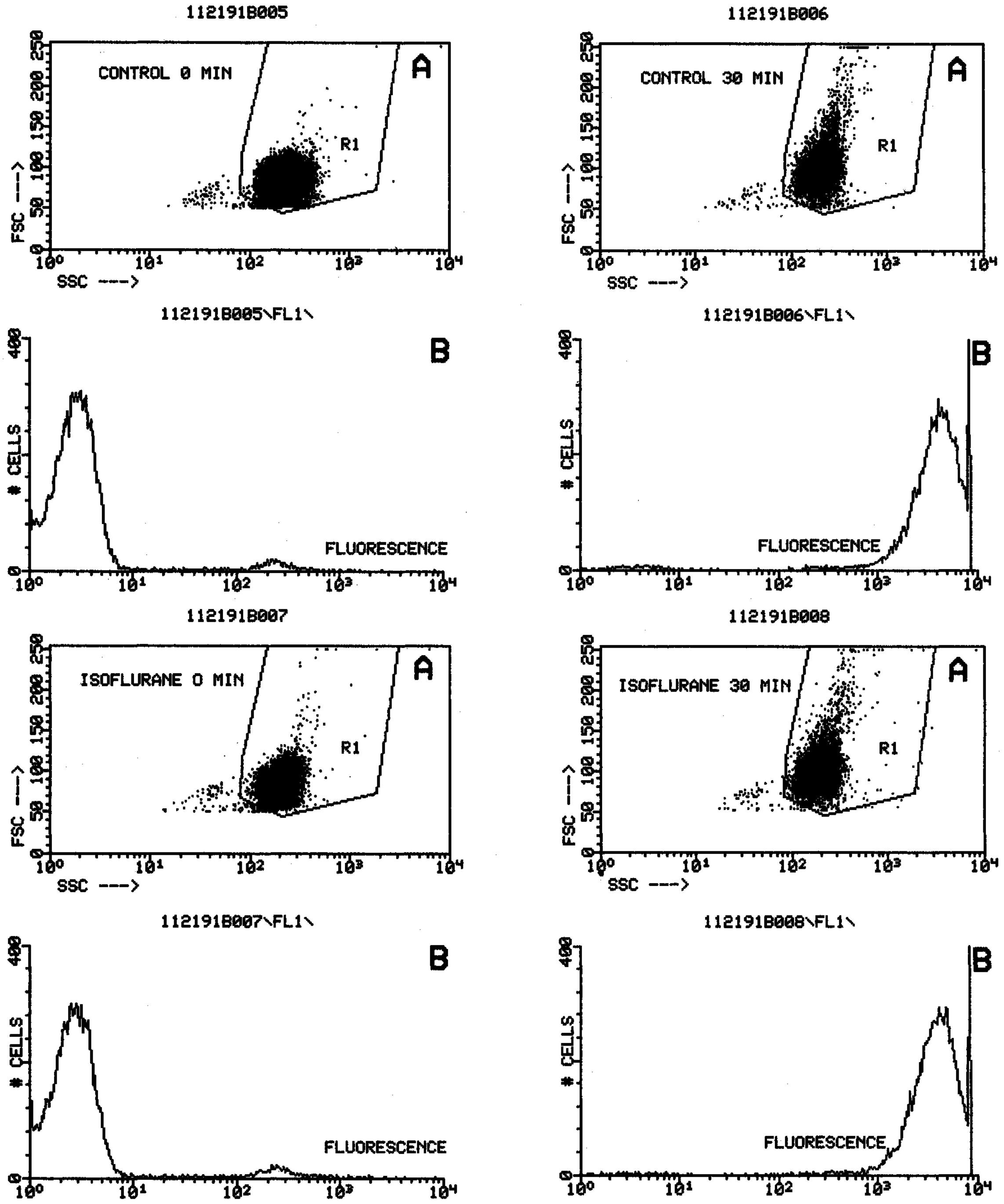

**Fig. 3.** Representative light scatter diagrams **(A)** showing forward (FSC) and 90° (SSC) side scatter and corresponding histograms of fluorescence intensity **(B)**, at 0 and 30 min of phagocytosis assay for control **(top 4 panels)** and isoflurane-treated **(bottom 4 panels)** neutrophils.

Therefore, bacterial infections occurring immediately after cesarean delivery do not appear to be the direct result of use of a general anesthetic such as isoflurane. The increased risk of infection is more likely due to the complex emergencies that create the need for general anesthesia for delivery.

### REFERENCES

1. Sweet RL, Ledger WJ: Puerperal infectious morbidity. Am J Obstet Gynecol 117:1093–1100, 1973.
2. Gibbs RS: Clinical risk factors for puerperal infections. Obstet Gynecol 55(Suppl):178S–184S, 1980.
3. Green SL, Sarubbi FA: Risk factors associated with post cesarean section febrile morbidity. Obstet Gynecol 49: 686–690, 1977.
4. Thomson DA: Anesthesia and the immune system. J Burn Care Rehabil 8:483–487, 1987.
5. Lippa S, De Sole P, Meucci E, Littaru GP, De Francisci G, Magalini SI: Effect of general anesthetics on human granulocyte chemiluminescence. Experientia 39:1386–1388, 1983.
6. Welch WD: Halothane reversibly inhibits human neutrophil bacterial killing. Anesthesiology 55:650–654, 1981.
7. Welch WD, Zaccari J: Effect of halothane and $N_2O$ on the oxidative activity of human neutrophils. Anesthesiology 57:172–176, 1982.
8. Nakagawara M, Takeshige K, Takamatsu J, Takahashi S, Yoshitake J, Minakami S: Inhibition of superoxide production and $Ca^{2+}$ mobilization in human neutrophils by halothane, enflurane, and isoflurane. Anesthesiology 64:4–12, 1986.
9. Welch WD: Effect of enflurane, isoflurane, and nitrous oxide on the microbicidal activity of human polymorphonuclear leukocytes. Anesthesiology 61:188–192, 1984.
10. Bassoe CF, Solberg CO: Phagocytosis of *Staphylococcus aureaus* by human leucocytes: Quantitation by a flow cytometric and microbiological method. Acta Pathol Microbiol Immunol Scand Sect C 92:43–50, 1984.
11. Bjerknes R, Bassoe CF, Sjursen H, Laerum OD, Solberg CO: Flow cytometry for the study of phagocyte functions. Rev Infect Dis 11:16–33, 1989.
12. Steinkamp JA, Wilson JS, Saunders GC, Stewart CC: Phagocytosis: Flow cytometric quantitation with fluorescent microspheres. Science 215:64–66, 1982.
13. Boyum A: Separation of leukocytes from blood and bone marrow. Scand J Clin Lab Invest 21(Suppl 97):77–89, 1968.
14. Gelfand JA, Fauci A, Green I, Frank M: A simple method for the determination of complement receptor bearing mononuclear cells. J Immunol 116:595–599, 1976.
15. Oda T, Maeda H: A new simple fluorometric assay for phagocytosis. J Immunol Methods 88:175–183, 1986.
16. Hed J, Hallden G, Johansson SGO, Larsson P: The use of fluorescence quenching in flow cytofluorometry to measure attachment and ingestion phases in phagocytosis in peripheral blood without prior cell separation. J Immunol Methods 101:119–125, 1987.
17. Moudgil GC, Allan RB, Russel RJ, Wilkinson PC: Inhibition by anesthetic agents, of human leucocytic locomotion towards chemical attractants. Br J Anaesth 49:97–104, 1977.
18. Moudgil GC, Gordon J, Forrest JB: Comparative effects of volatile anesthetic agents and nitrous oxide on human leucocyte chemotaxis in vitro. Can Anaesth Soc J 31:631–637, 1984.
19. Erskine R, James MFM: Isoflurane but not halothane stimulates neutrophil chemotaxis. Br J Anaesth 64:723–727, 1990.
